# Design, Synthesis and Biological Evaluation of Novel Pyrimido[4,5-*d*]pyrimidine CDK2 Inhibitors as Anti-Tumor Agents

**DOI:** 10.3797/scipharm.1103-16

**Published:** 2011-05-08

**Authors:** Samir M. El-Moghazy, Diaa A. Ibrahim, Nagwa M. Abdelgawad, Nahla A. H. Farag, Ahmad S. El-Khouly

**Affiliations:** 1 Department of Pharmaceutical Chemistry, Faculty of Pharmacy, Cairo University, Cairo, Egypt; 2 Department of Organic Chemistry, National Organization for Drug Control and Research, P.O. Box 29, Cairo, Giza 12311, Egypt; 3 Pharmaceutical Chemistry Department, Faculty of Pharmacy, Misr International University, Km 28 Cairo-Ismailia Road, Cairo, Egypt

**Keywords:** Drug Design, Pharmacophore, Pyrimido[4,5-*d*]pyrimidine, CDK2, Anti-tumor

## Abstract

A series of 2,5,7-trisubstituted pyrimido[4,5-*d*]pyrimidine cyclin-dependent kinase (CDK2) inhibitors is designed and synthesized. 6-Amino-2-thiouracil is reacted with an aldehyde and thiourea to prepare the pyrimido[4,5-*d*]-pyrimidines. Alkylation and amination of the latter ones give different amino derivatives. These compounds show potent and selective CDK inhibitory activities and inhibit in vitro cellular proliferation in cultured human tumor cells.

## Introduction

Conventional anti-cancer drugs such as alkylating agents, antimetabolites, topoisomerase inhibitors, and anti-microtubule agents have traditionally focused on targeting DNA synthesis and cell division. Although these drugs show efficacy, their lack of selectivity for tumor cells over normal cells usually lead to severe adverse effects such as bone marrow suppression, cardiac, hepatic, and renal toxicities which limit their use. In an attempt to circumvent these unpleasant side effects, a new class of anti-cancer agents known as signal transduction or secondary message inhibitors has been developed [1]. Cells use a wide variety of both intra- and intercellular mechanisms to signal for processes including growth, apoptosis, and intracellular protein degradation. Due to up-regulation or greater dependence on some of these pathways in tumor cells, inhibition should lead to anti-cancer effects [2].

The D-type cyclins and their kinase partners (CDKs) have an important role in cell cycle as they phosphorylate the tumor suppressor protein, retinoblastoma protein (PRB) during the G-1 phase of the cell cycle and contribute in its inactivation. They also act as integrators of extracellular signals. Specific CDKs operate in distinct phases of the cell cycle e.g. CDK4/CyclinD and CDK6/CyclinD are responsible for the progression through the G1 phase, CDK2/CyclinE is required for the progression from G1 phase to S, CDK2/CyclinA is required for the transition through S, while CDK1/CyclinB is required for the G2/M transition [1–4]. These CDK-cyclin complexes are in turn regulated by small inhibitory proteins called Endogenous CDK inhibitors.

Pyrimidopyrimidines are annelated uracils that have attracted considerable interest in recent years [5, 6]. Their derivatives have been known to display a wide range of pharmacological activities, and their potent inhibitory properties regarding the tyrosine kinase domain of epidermal growth factor receptor [7], 5-phosphoribosyl-1-pyrophosphate synthetase [8] and dihydrofolate reductase [9] have been fully demonstrated. Numerous reports delineate the anti-tumour [10], antiviral [10, 11], antioxidant [12], antifungal [13] and hepatoprotective activities of these compounds [14].

In our program to develop CDK2 inhibitors as anti-cancer agents, we recently reported that pyrazolo[3,4-*d*]pyrimidines and 3,6-disubstituted [1,2,4]triazolo[3,4-*b*][1,3,4]thiadiazole analogs are novel anti-cancer inhibitors and anti-proliferative agents [15, 16]. To discover structurally different CDK2 inhibitors with improved potency and selectivity, we have designed, synthesized, and evaluated trisubstituted pyrimido[4,5-*d*]pyrimidines as inhibitors of tumor cell proliferation.

## Results and Discussion

### Rational and design

We have been interested in developing inhibitors of CDK2, and the crystal structure of the kinases domain has been solved by X-ray crystallography. These targets therefore were not only amenable to hit discovery by virtual screening techniques but also suited to show our pharmacophore models to design a new lead compounds that can act as CDK2 inhibitors with reasonable selectivity. We selected pyrimido[4,5-*d*]pyrimidine moiety to mimic the adenine region at ATP binding site and according to modeling results we optimized the side chains to improve the activity and selectivity.

#### Preparation of the designed structure

The coordinate for the protein structure was obtained from the RCSB Protein Data Bank (PDB) (1ke6) [17]. Protein Structure was prepared using Discovery Studio (DS 2.0) software package [18]. The invalid or missing residues were added and the structures were aligned using the protein structure alignment module. Hydrogen atoms were added and the structure was minimized using CHARMm force field to relax the backbone and to remove the clashes. The protein was inspected visually for accuracy in the X2 dihedral angle of Asn, His residues and the X3 angle of Gln.

The proposed compounds were optimized by semiemperical method (AM1) using Chem3D to eliminate bond length and bond angle biases then saved to be used in the pharmacophore mapping step.

#### Pharmacophore Generation

**Fig. 1.**
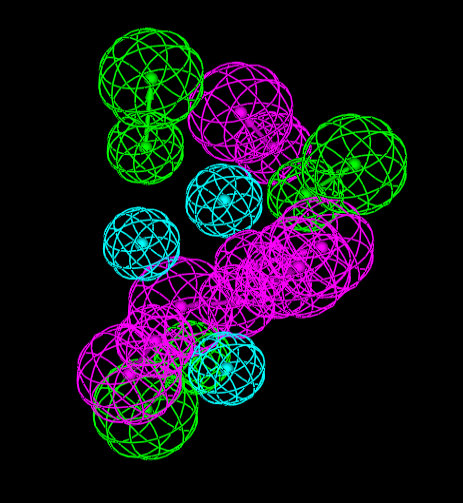
Pharmacophore model (1) derived from CDK2 crystal structure (PDB code: 1ke6) (Green, acceptor atom; Magenta, donor atom, Cyan, hydrophobic centers)

Two pharmacophore models were generated. The first was more accurate specific cyclin-dependant kinase pharmacophore model and derived from the PDB (1ke6) crystal structure. We defined the active pocket features (all hydrogen bond donors, acceptors and hydrophobic centers) and according to these features we estimate the first pharmacophore model. In this pharmacophore model (Fig 1) a donor atom associated with the protein donor site is used to donate the key hydrogen bond interaction.

**Fig.2.**
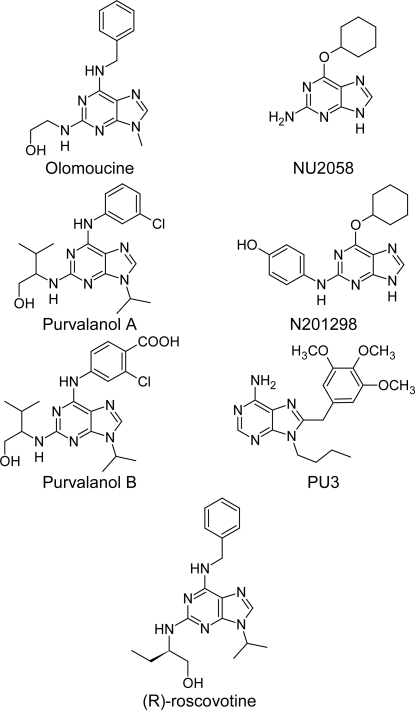
CDK2 inhibitors which were used for building the pharmacophore model (2)

**Fig. 3.**
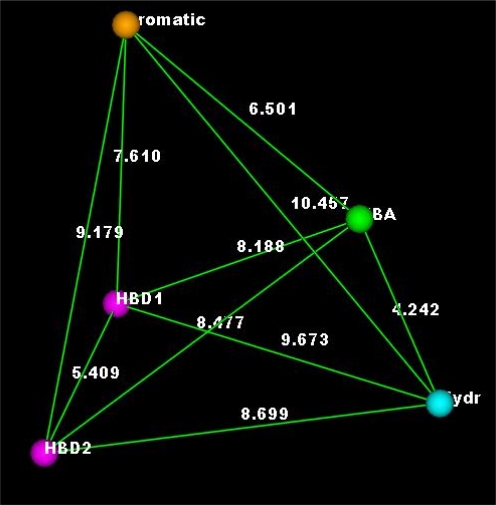
Pharmacophore model 2, which derived from potential CDK2 inhibitors and used in the pre-selection of the proposed compounds (Hydr, hydrophobic center; HBA, acceptor atom; HBD, donor atom; Aromatic, aromatic center).

The second pharmacophore model was derived from 7 potent inhibitors (Fig 2) of CDK2 by calculating the common features protocol. This model was made up of hydrophobic center, aromatic ring, acceptor atom associated to its protein donor site and donor atom (Fig 3).

The interfeature distances were considered to be 7.61, 6.50, 4.24, 8.69 and 5.4 A° for the distances between the aromatic center and the donor 1, the aromatic center and the acceptor, the acceptor and hydrophobe, the hydrophobe and the donor 2, and the donor 2 to donor 1, respectively. The association of the acceptor atom to the donor site in the protein ensured the overall orientation of the molecules with respect to the kinase. Only one angle constraint was used for the hydrophobic and the acceptor atom features, thus allowing the hydrophobic and the aromatic centers to cover the large domain in the kinase active site, from the hydrophobic to the sugar pocket. Since not all proposed compounds place hydrophobes in both regions, a partial match directive was used on the query for the hydrophobic centers to match compounds that contain only one. We built the first pharmacophore model to ensure that proposed compounds with different interaction modes (different from interaction modes of compounds in Fig 2) will be taken in consideration in this model.

**Fig. 4.**
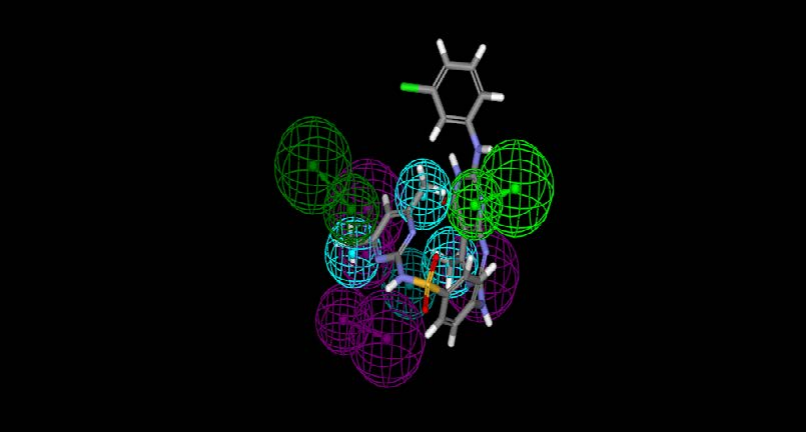
**M**apping of compound **7f** to pharmacophore 1 (Fit value = 4.1)

**Fig. 5.**
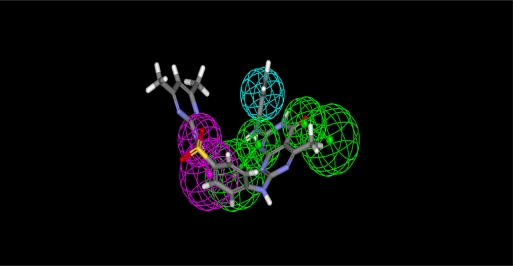
Mapping of compound **7f** to pharmacophore 2 (Fit value = 2.95)

Using these pharmacophore models, we mapped our proposed compounds which contain pyrimido[4,5-*d*]pyrimidine moiety to the generated pharmacophore models in order to find the promising compounds that are capable of binding to CDK2 with a similar set of interactions (Figs 4 and 5). Finally, we selected the proposed compounds with high fit values for synthesis.

### Chemistry

Pietro Biginelli reported the first synthesis of 3,4-dihydropyrimidin-2(1*H*)-ones by a very simple one-pot condensation reaction of aromatic aldehyde, urea and ethyl acetoacetate in ethanolic solution [19] and as a new modification we prepared pyrimido[4,5-*d*]pyrimidine derivatives by using 6-amino thiouracil derivative, aldehydes and guanidine derivatives. The best reaction product was achieved by using glacial acetic acid as a solvent.

The newly synthesized compounds were prepared as outlined in Scheme 1; Compound **1** was prepared by the reaction of thiourea with ethyl cyanoacetate in refluxing sodium ethoxide for 3 hours in 95% yield as reported before [20]. Alkylation of mercaptopyrimidine **1** was carried out by a standard alkylation method using sodium hydroxide solution (2N) as base at room temperature [20].

To facilitate the synthetic pathways, we prepared the guanidine derivatives **5a,b** by reaction of isothiurinium HCl with sulfadimidine and 1-(4-aminophenyl)-3-phenylthiourea, which were prepared by the reaction of 4-nitroaniline with phenylisothiocyanate [21] followed by the reduction of the thiourea derivative with zinc and ammonium formate in ethanol at reflux. The base was obtained by trituration with NaHCO_3_ solution (Scheme 2).

As a new modification of the Biginelli reaction, we synthesized compounds of type **6a–d** by the reaction of aminopyrimidone **2** with aldehydes and guanidine derivatives **5a**,**b** in refluxing glacial acetic acid for 6–8 hours in up to 80% yield, which reacted with different amines in refluxing dimethyl formamide to afford the bicyclic products **7a–i** (Scheme 3).

### Biology

#### Biochemical Assay

CDK2, CDK4 and EGFR inhibitory activities of pyrimido[4,5-*d*]pyrimidines prepared above were shown in Table 1, together with those of roscovitine [22, 23] and PYK2104 as reference compounds. CDK2 is one of CDK family protein which has a proline directed serine/threonine kinase activity whereas EGFR belongs to receptor tyrosine kinase family. Compounds were further evaluated for their cell division inhibitory activities against 60 human tumor cell lines. The newly synthesized compounds were tested against CDK4 because CDK4 shares CDK2 the essential function of coupling the G1/S transition with mitosis [24].

Inhibitory activities are given as IC_50_ values; for less active compounds percentage inhibition at a concentration of 10 μM is shown. It is noteworthy that most compounds with high activities towards CDK2 (IC_50_ < 1 μM) proved to be selective inhibitors. The data presented in Table 1 clearly showed that pyrimido[4,5-*d*]pyrimidine derivatives could be a good inhibitors of the CDK2, compound **7f** is more active than roscovitine the reference with IC_50_ 0.05. Two compounds **7e** and **7a** showed a very good inhibition with IC_50_ values 0.25 and 0.31 μM respectively. Compound **7d** had the least selectivity towards the CDK2 inhibition and the other compounds showed moderate activity and selectivity. Compounds **6d** and **7f** showed the best CDK4 activity with IC_50_ 0.66 and 0.86 respectively. Four compounds **6a**, **7c**, **7h** and **7i** showed no activity on CDK4. The other compounds showed moderate activity and selectivity except compounds **7d** and **7g** which had very poor activity. Finally, most compounds did not show any significant EGFR inhibitory activity, indicating a good selectivity in the situation of protein kinase inhibitors.

According to the data in table 1 we can say that the structural postulates for potential pyrimido[4,5-*d*]pyrimidine inhibitors derived from Roscovitine- or ATP-type binding model are proved to be correct.

#### Cytotoxicity assay

Evaluation of anti-cancer activities on pyrimido[4,5-*d*]pyrimidine derivatives were performed at the National Cancer Institute (NCI) USA. First the newly synthesized compounds have been evaluated in primary anti-cancer assay at 10^−5^M concentration against 60 human tumor cell lines then the active compounds were tested at 5 different concentrations against the same 60 human tumor cell lines. The biological data for the newly synthesized compounds are compiled in table 2

By the analysis of the data in table 2 we found that all the tested compounds showed different activities on the different cell lines. Compounds **6a**, **6c** and **7e** had cell death activities on Non-small cell lung cancer (HOP-92) 102.6% inhibition, Renal cancer (RXF 393) 124% and 112.9% inhibition respectively. Compounds 7c and 7d showed very high growth inhibitions on Melanoma (UACC-257) 71.36% and 79.49 growth inhibition % respectively. Compounds **7a**, **7b**, **7c**, **7f** and **7h** showed high growth inhibitions ranging from 50 to 70 % on Non-small cell lung cancer, Ovarian cancer, Leukemia, and Melanoma. The rest of compounds and some of the aforementioned compounds had moderate to low growth inhibition ranging from 20 to 50 % in different tumor cell lines.

It seems that there is no good correlation between in-vitro CDK2 inhibitory activity and cell growth inhibition activity among some tested compounds e.g. compound **7f** is the most active inhibitor against kinases but it does not seem to be the most potent against cell lines. Currently the reason is not clear but it may be due to different abilities to internalize inside cells.

## Conclusions

All the biological results were complied with the molecular modeling studies to a considerable extent. The experimental activity of some compounds was found to be different from the expected theoretical values, some compounds showed high theoretical value while they had moderate to low biological results with respect to the other compounds, which can be explained by the conformational analysis of the synthesized compounds or the difficulty in cell permeability.

## Experimental

### Chemistry

All melting points were uncorrected and determined by the open capillary method using a Gallenkamp melting point apparatus. IR spectra were recorded (KBr) on a Pye-Unicam SP-883 Perkin Elmer spectrophotometer. ^1^HNMR spectra were recorded on a varian EM 400–600 MHz spectrometer using DMSO-d*_6_* as a solvent and TMS as an internal reference, chemical shifts are expressed in δ units (ppm). Mass spectra were recorded with a mass spectrometer MS9 (AEI) 70 ev. All the analytical data were obtained from Microanalytical Data Unit at Cairo University and Toledo University. All the results were within an acceptable range.

### 6-Amino-2-sulfanylpyrimidin-4(3*H*)-one (1)

Compound 1 was prepared according to a literature method [20].

### 6-Amino-2-(benzylsulfanyl)pyrimidin-4(3*H*)-one (2)

#### Method A

To a solution of compound 1 (14.3 g, 0.1 mol) in a mixture of dimethyl sulfoxide (20 mL) and sodium hydroxide 2N (5 mL), Benzyl chloride (11.6 mL, 0.1 mol) was added drop-wise with stirring for one and half hour. The resulting mixture was poured onto cold water (200 mL), acidified by glacial acetic acid then filtered using vacuum filtration, air dried and recrystallized from alcohol.

#### Method B

To a solution of compound 1 (14.3 g, 0.1 mol) in a mixture of dimethyl formamide (20 mL), and potassium carbonate (55.3 g, 0.4 mol), Benzyl chloride (11.6 mL, 0.1mol) was added drop-wise with stirring overnight. The resulting reaction mixture was poured onto cold water (200 mL), acidified by glacial acetic acid then filtered using vacuum filtration, dried in air and recrystallized from alcohol.

Yellowish white crystals (95% yield); m.p. 122–124 °C; ^1^HNMR (DMSO-d_6_): δ ppm 11.1 (s, 1H, NH), 8.5 (s, 2H, NH_2_), 7.5–7.3 (m, 5H, Ar-H), 6.6 (s, 1H, H5), 4.5 (s, 2H, SCH_2_). MS m/z: 233.3 (M^+^). Anal. Calcd for C_11_H_11_N_3_OS: C, 56.63; H, 4.75; N, 18.01 Found: C, 56.48; H, 4.73;N. 17.91.

### 1-(4-Nitrophenyl)-3-phenylthiourea (3)

To a solution of 4-nitroaniline (1.4 g, 0.01 mol) in ethanol (20 mL) was added an equimolar amount of phenylisothiocyanate (1.35 g, 0.01 mol) then Potassium hydroxide (1.7g, 0.03 mol) was added. The reaction mixture was stirred for 4h at R. T. then the separated solid product was filtered off, dried, and recrystallized from ethanol to give compound **3**.

Yellow crystals (94% yield); m.p. 118–120 °C; ^1^HNMR (DMSO-d_6_): δ ppm 12.4 (s, 1H, SH), 9.5 (s, 2H, 2NH), 8.2–8.0 (m, 2H, Ar-H), 7.4–7.2 (m, 5H, Ar-H), 7.0–6.8 (m, 2H, Ar-H). MS m/z: 273 (M^+^). Anal. Calcd for C_13_H_11_N_3_O_2_S: C, 57.13; H, 4.06; N, 15.37 Found: C, 57.48; H, 4.13; N. 15.71.

### 1-(4-Aminophenyl)-3-phenylthiourea (4)

A suspension of nitrophenylthiourea derivative **3** (1.4 g, 5 mmol) and Zn dust (0.4 g, 6 mmol) in methanol (15 ml) was stirred with ammonium formate (0.5 g) at room temperature. After completion of the reaction (monitored by TLC), the mixture was filtered off. The organic layer was evaporated and the residue dissolved in CHCl_3_ and washed with saturated NaCl to remove ammonium formate. The organic layer upon evaporation gave the desired amino derivative **4**.

Yellowish white crystals (86% yield); m.p. 134–136 °C; ^1^HNMR (DMSO-d_6_): δ ppm 12.1 (s, 1H, SH), 8.9 (s, 2H, 2NH), 7.5–7.1 (m, 5H, Ar-H), 6.9–6.7 (m, 4H, Ar-H), 6.4 (s, 2H, NH_2_). MS m/z: 243 (M^+^). Anal. Calcd for C_13_H_13_N_3_S: C, 64.17; H, 5.39; N, 17.27 Found: C, 64.48; H, 5.13; N. 17.71.

### Arylguanidine derivatives (5)

A mixture of benzyl isothiourinium hydrochloride (10.2 g, 0.05 mol) and the appropriate amine (0.06 mol) in absolute alcohol (200 mL) was refluxed for 16 hours. The reaction mixture was evaporated to dryness in a water bath and the resulting precipitate was triturated with NaHCO_3_ solution (100 mL, 2%). The resulting precipitate was filtered, washed thoroughly with water, air dried and used without further purification for the next step.

#### 1-(4-Guanidinophenyl)-3-phenylthiourea (**5a**)

Brown crystals (89% yield); m.p. 140–142 °C; ^1^HNMR (DMSO-d_6_): δ ppm 9.1 (s, 2H, 2NH), 7.6–7.4 (m, 4H, Ar-H), 7.3–7.2 (m, 5H, Ar-H), 7.1 (s, 1H, NH), 6.9 (s, 1H, NH), 6.7 (s, 2H, NH_2_). MS m/z: 287 (M^+2^). Anal. Calcd for C_14_H_15_N_5_S: C, 58.92; H, 5.30; N. 24.54 Found: C, 59.48; H, 4.73;N. 23.71.

#### *N*-(4,6-Dimethylpyrimidin-2-yl)-4-guanidinobenzenesulfonamide (**5b**)

White crystals (79% yield); m.p. 220–222 °C; ^1^HNMR (DMSO-d_6_): δ ppm 8 (s, 1H, SO_2_NH), 7.8–7.7 (dd, 2H, Ar-H), 7.4–7.3 (dd, 2H, Ar-H), 7.1 (s, 1H, NH), 7 (s, 1H, H5-pyrimidine), 6.8 (s, 1H, NH), 6.7 (s, 2H, NH_2_), 2.1 (s, 6H, 2CH_3_). MS m/z: 321 (M^+1^). Anal. Calcd for C_13_H_16_N_6_O_2_S: C, 48.74; H, 5.03; N, 26.23 Found: C, 49.48; H, 4.73; N, 25.41.

### 2-(Benzylsulfanyl)-5-aryl-7-(arylamino)pyrimido[4,5-*d*]pyrimidin-4(3*H*)-ones (6)

A solution of compound 5a,b (0.05 mol) and the appropriate aldehyde in glacial acetic acid was refluxed for an hour, and then compound 2 (11.7 g, 0.05 mol) was added and refluxed for another 10 hours. The reaction mixture was concentrated to half volume, cooled, poured on cold water (500 mL), filtered using vacuum filtration, air dried and recrystallized from DMF-H_2_O.

#### 1-(4-{[7-(Benzylsulfanyl)-4-(2,4-dihydroxyphenyl)-5-oxo-5,6-dihydro-pyrimido[4,5-*d*]pyrimidin-2-yl]amino}phenyl)-3-phenylthiourea (**6a**)

Brown red crystals (80% yield); m.p. > 300 °C; ^1^HNMR (DMSO-d_6_): δ ppm 11.9 (s,1H, NH pyrimidone), 8.6 (s, 2H, NHCSNH), 8.4 (s, 2H, 2OH), 8.3 (s, 1H, NH), 7.4–6.8 (m, 17H, Ar-H), 3.7 (s, 2H, SCH_2_). ^13^C NMR (DMSO-d_6_, 100 MHz): δ ppm 38, 104, 106.1, 112.2, 116.3, 123.3, 128.5, 130.6, 132.2, 133.4, 135.8, 137.1, 138.6, 140.1, 142.5, 144.4, 145.9, 146.8, 150.1, 160.2, 162.4, 164.4, 165.8, 167.7, 176.2, 181.8. MS m/z: 620 (M^+1^). Anal. Calcd for C_32_H_25_N_7_O_3_S_2_: C, 62.02; H, 4.07; N. 15.82 Found: C, 62.48; H, 3.73; N. 15.51.

#### 4-{[7-(Benzylsulfanyl)-4-(2,4-dihydroxyphenyl)-5-oxo-5,6-dihydropyrimido[4,5-*d*]pyrimidin-2-yl]amino}-*N*-(4,6-dimethylpyrimidin-2-yl)benzenesulfonamide (**6b**)

Orange red crystals (75% yield); m.p. > 300 °C; ^1^HNMR (DMSO-d_6_): δ ppm 11.5 (s, 1H, NH pyrimidone), 8.9 (s, 2H, 2OH), 8.7 (s, 2H, 2NH), 7.6–7.0 (m, 9H, Ar-H), 6.9 (s, 1H, H5 pyrimidine), 4.4 (s, 2H, SCH_2_), 2.2 (s, 6H, 2CH_3_). MS m/z: 656 (M^+1^). Anal. Calcd for C_31_H_26_N_8_O_5_S_2_: C, 56.87; H, 4.00; N. 17.11. Found: C, 56.11; H, 4.15; N. 16.89.

#### 1-(4-{[7-(Benzylsulfanyl)-4-methyl-5-oxo-5,6-dihydropyrimido[4,5-*d*]pyrimidin-2-yl]amino}-phenyl)-3-phenylthiourea (**6c**)

Purple crystals (80% yield); m.p. > 300 °C; ^1^HNMR (DMSO-d_6_): δ ppm 12.3 (s,1H, NH pyrimidone), 9.2 (s, 2H, NHCSNH), 8.8 (s, 1H, NH), 7.8–7.1 (m, 14H, Ar-H), 3.9 (s, 2H, SCH_2_), 2.5 (s, 3H, CH_3_). MS m/z: 525 (M^+^). Anal. Calcd for C_27_H_23_N_7_OS_2_: C, 61.69; H, 4.41; N.18.65. Found: C, 60.73; H, 4.34; N. 19.02.

#### 4-{[7-(Benzylsulfanyl)-4-methyl-5-oxo-5,6-dihydropyrimido[4,5-*d*]pyrimidin-2-yl]amino}-*N*-(4,6-dimethylpyrimidin-2-yl)benzenesulfonamide (**6d**)

Pale yellow crystals (70% yield); m.p. > 300 °C; ^1^HNMR (DMSO-d_6_): δ ppm 11.1 (s, 1H, NH pyrimidone), 8.9 (s, 2H, 2NH), 7.8–7.3 (m, 9H, Ar-H), 7.2 (s, 1H, H5 pyrimidine), 4.0 (s, 2H, SCH_2_), 2.6 (s, 3H, CH_3_), 2.1 (s, 6H, 2CH_3_). ^13^C NMR (DMSO-d_6_, 100 MHz): δ ppm 20.5, 23.8, 36.9, 108.2, 115.2, 123.5, 129.1, 130.9, 132.5, 135.3, 139.1, 140.2, 145.7, 152.2, 155.1, 163.1, 165.9, 167.8, 169.3, 172.5. Anal. Calcd for C_26_H_24_N_8_O_3_S_2_: C, 55.7; H, 4.31; N. 19.99. Found: C, 54.82; H, 4.19; N. 20.27.

### 7-(Alkyl/Arylamino)-5-alkyl-2-(arylamino)pyrimido[4,5-*d*]pyrimidin-4(3*H*)-one (7)

To a solution of Compound **6a–d** (5 mmol) in a mixture of dimethyl formamide (10 mL) and glacial acetic acid (5 mL), the appropriate amine (5 mmol) was added. The reaction mixture was refluxed for 8 hours, cooled and poured onto cold water (200 mL). The resulting precipitate was collected, air dried and recrystallized from acetic acid-H_2_0.

#### 1-(4-{[7-(Benzylamino)-4-(2,4-dihydroxyphenyl)-5-oxo-5,6-dihydropyrimido[4,5-*d*]pyrimidin-2-yl]amino}phenyl)-3-phenylthiourea (**7a**)

Brown red crystals (60% yield); m.p. > 300 °C; ^1^HNMR (DMSO-d_6_): δ ppm 9.9 (s, 1H, NH pyrimidone), 8.6 (s, 2H, 2OH), 8.2 (s, 2H, HNCSNH), 6.8–7.3 (m, 17H, Ar-H), 6.9 (s, 1H, NH Benzyl), 6.5 (s, 1H, NH), 3.8 (s, 2H, PhCH_2_). ^13^C NMR (DMSO-d_6_, 100 MHz): δ ppm 40.3, 98.9, 101.6, 106.4, 110.7, 117.5, 120.8, 123.7, 124.9, 125.5, 127.6, 128.9, 130.1, 131.9, 132.7, 135.5, 136.4, 139.1, 152.1, 155.9, 158.5, 160.3, 161.6, 163.8, 171.4, 181.8. IR (KBr) cm^−1^: 3296, 3182, 3057, 2922, 1627, 1593. MS m/z: 604 (M^+1^). Anal. Calcd for C_32_H_26_N_8_O_3_S: C, 63.77; H, 4.35; N, 18.59. Found: C, 64.24; H, 4.12; N, 18.13.

#### 1-(4-{[7-(Benzylamino)-4-methyl-5-oxo-5,6-dihydropyrimido[4,5-*d*]pyrimidin-2-yl]amino}-phenyl)-3-phenylthiourea (**7b**)

Purple crystals (65% yield); m.p. > 300 °C; ^1^HNMR (DMSO-d_6_): δ ppm 10.1 (s, 1H, NH pyrimidone), 8.5 (s, 2H, HNCSNH), 7.2–7.8 (m, 14H, Ar-H), 6.9 (s, 1H, NH Benzyl), 6.6 (s, 1H, NHPh), 4.3 (s, 2H, PhCH_2_), 2.3 (s, 3H, CH_3_). IR (KBr) cm^−1^: 3296, 3182, 3055, 2924, 1631, 1593. Anal. Calcd for C_27_H_24_N_8_OS: C, 63.76; H, 4.76; N, 22.03. Found: C, 63.64; H, 4.31; N, 22.84.

#### 4-{[7-(Benzylamino)-4-methyl-5-oxo-5,6-dihydropyrimido[4,5-*d*]pyrimidin-2-yl]amino}-*N*-(4,6-dimethylpyrimidin-2-yl)benzenesulfonamide (**7c**)

Yellow crystals (50% yield); m.p. > 300 °C; ^1^HNMR (DMSO-d_6_): δ ppm 11.5 (s, 1H, NH pyrimidone), 7.4 (s, 1H, NH Benzyl), 7.3–7.1 (m, 9H, Ar-H), 6.5 (s, 1H, H5 pyrimidine), 4.9 (s, 1H, SO_2_NH), 4.3 (s, 2H, PhCH_2_), 2.8 (s, 3H, CH_3_), 2.2 (s, 6H, 2CH_3_). IR (KBr) cm^−1^: 3311, 3199, 3026, 2922, 1630, 1570. Anal. Calcd for C_32_H_26_N_8_O_3_S: C, 57.45; H, 4.64; N, 23.19. Found: C, 57.82; H, 4.38; N, 23.56.

#### 1-[4-({7-[(3-Chlorophenyl)amino]-4-(2,4-dihydroxyphenyl)-5-oxo-5,6-dihydro-pyrimido[4,5-*d*]pyrimidin-2-yl}amino)phenyl]-3-phenylthiourea (**7d**)

Brown red crystals (55% yield); m.p. > 300 °C; ^1^HNMR (DMSO-d_6_): δ ppm 11.2 (s, 1H, NH pyrimidone), 8.8 (s, 2H, NHCSNH), 8.2 (s, 2H, 2OH), 8.1 (s, 2H, 2NH), 7.3–-6.9 (m, 16H, Ar-H). IR (KBr) cm^−1^: 3360, 3340, 3260, 3220, 3026, 2970, 1640, 1590. MS m/z: 525 (M^+1^). Anal. Calcd for C_31_H_23_ClN_8_O_3_S: C, 59.76; H, 3.72; N, 17.98. Found: C, 60.03; H, 3.92; N, 18.35.

#### 1-[4-({7-[(3-Chlorophenyl)amino]-4-methyl-5-oxo-5,6-dihydropyrimido[4,5-*d*]pyrimidin-2-yl}amino)phenyl]-3-phenylthiourea (**7e**)

Purple crystals (55% yield); m.p. > 300 °C; ^1^HNMR (DMSO-d_6_): δ ppm 11.5 (s, 1H, NH pyrimidone), 8.9 (s, 2H, NHCSNH), 8.5 (s, 2H, 2NH), 7.3–6.9 (m, 13H, Ar-H), 2.2 (s, 3H, CH_3_).^13^C NMR (DMSO-d_6_, 100 MHz): δ ppm 19.8, 102.6, 120.1, 121.9, 122.4, 123.1, 125.4, 126.9, 127.7, 130.1, 131.8, 132.5, 135.8, 140.1, 141.6, 142.9, 144.1, 160.3, 164.2, 167.9, 172.2, 180.8. IR (KBr) cm^−1^: 3300, 3197, 3055, 2924, 1631, 1593. MS m\z: 530 (M^+1^). Anal. Calcd for C_26_H_21_ClN_8_OS: C 59.03; H, 4.00; N, 21.18. Found: C, 59.64; H, 3.89; N, 21.34.

#### 4-({7-[(3-Chlorophenyl)amino]-4-methyl-5-oxo-5,6-dihydropyrimido[4,5-*d*]pyrimidin-2-yl}amino)-N-(4,6-dimethylpyrimidin-2-yl)benzenesulfonamide (**7f**)

Yellow crystals (65% yield); m.p. > 300 °C; ^1^HNMR (DMSO-d_6_): δ ppm 11.6 (s, 1H, NH pyrimidone), 8.8 (s, 1H, SO_2_NH), 8.4 (s, 2H, 2NH), 7.4–7.2 (m, 8H, Ar-H), 6.8 (s, 1H, H5 Pyrimidine), 2.7 (s, 3H, CH_3_), 2.1(s, 6H, 2CH_3_). IR (KBr) cm^−1^: 3448, 3142, 3026, 2924, 1620, 1570. Anal. Calcd for C_25_H_22_ClN_9_O_3_S: C, 53.24; H, 3.93; N, 22.35. Found: C, 53.19; H, 3.82; N, 22.74.

#### 4-{[5-(2,4-Dihydroxyphenyl)-4-oxo-7-({4-[(phenylcarbamothioyl)amino]phenyl}amino)-3,4-dihydropyrimido[4,5-*d*]pyrimidin-2-yl]amino}benzenesulfonic acid (**7g**)

Reddish brown crystals (62% yield); m.p. > 300 °C; IR (KBr) cm^−1^: 3296, 3140, 3057, 2922, 1629, 1593; ^1^HNMR (DMSO-d_6_): δ ppm 12.1 (s, 1H, NH pyrimidone), 11.1 (s, 1H, OH), 10.5 (br, 4H, NH or SH), 10.2 (s,2H,2OH), 6.9–7.8 (m, 16H, Ar-H); MS m/z: 669 (M^+^). Anal. Calcd for C_31_H_24_N_8_O_6_S_2_: C, 55.68; H, 3.62; N, 16.76. Found: C, 56.03; H, 3.74; N, 17.21

#### 4-{[5-Methyl-4-oxo-7-({4-[(phenylcarbamothioyl)amino]phenyl}amino)-3,4-dihydropyrimido[4,5-*d*]pyrimidin-2-yl]amino}benzenesulfonic acid (**7h**)

Purple crystals (60% yield); m.p. > 300 °C; IR (KBr) cm^−1^: 3311, 3199, 3026, 2922, 1630, 1570; ^1^HNMR (DMSO-d_6_): δ ppm 11.8 (s, 1H, NH pyrimidone), 11.0 (s, 1H, OH), 10.8 (br, 4H, NH or SH), 7.2–8.1 (m, 13H, Ar-H), 2.8 (s, 3H, CH_3_); MS m/z: 575 (M^+1^). Anal. Calcd for C_26_H_22_N_8_O_4_S_2_: C, 54.34; H, 3.86; N, 19.50. Found: C, 54.67; H, 3.34; N, 20.11.

#### 4-{[7-({4-[(4,6-Dimethylpyrimidin-2-yl)sulfamoyl]phenyl}amino)-5-methyl-4-oxo-3,4-dihydro-pyrimido[4,5-*d*]pyrimidin-2-yl]amino}benzenesulfonic acid (**7i**)

Purple crystals (65% yield); m.p. > 300 °C; ^1^HNMR (DMSO-d_6_): δ ppm 12.1 (s, 1H, NH pyrimidone), 10.2 (s, 1H, SO_2_NH), 10.1 (s, 2H, 2NH), 10.0 (s, 1H, OH), 7.8–7.3 (m, 8H, Ar-H), 7.1 (s, 1H, H5 Pyrimidine), 2.6 (s, 3H, CH_3_), 2.2 (s, 6H, 2CH_3_). ^13^C NMR (DMSO-d_6_, 100 MHz): δ ppm 20.6, 22.5, 102.6, 110.2, 118.3, 120.5, 124.6, 125.6, 134.1, 138.3, 140.2, 142.6, 147.6, 150.1, 158.7, 160.4, 162.9, 165.3, 170.1. MS m/z: 6115 (M^+1^). Anal. Calcd for C_25_H_23_N_9_O_6_S_2_: C, 49.25; H, 3.80; N, 20.68. Found: C, 49.74; H, 3.98; N, 21.21.

### Biological Methods

#### Enzymatic activity inhibition assay

The inhibition studies of cell cycle dependent kinase 2 were performed for the synthesized compounds along with roscovitine as reference compound. We synthesized roscovitine according to reported method [25]. CDK2/cyclinA enzyme was purified from infected sf21 insect cells. For baculoviral overexpression of proteins, we sub-cloned human CDK2 c-DNA tagged by hexa-histidine on its N-terminal and human cyclinA c-DNA into pBacPak 8 expression vector, respectively. Baculovirus which carries each gene was generated using baculovirus generating kit. CDK2/cyclinA enzyme was purified using Ni^+2^-affinity resin from sf21 insect cell culture into which CDK2 and cyclin A carrying baculoviruses were cotransfected. Enzyme assays were done in 20 mL of 50 mMTris-HCl containing 10 mM ATP, 0.2 mCi of gamma-P^32^ ATP, 10 mM MgCl_2_, 5 mM DTT and 4 mg of histone H1 was used as a substrate. The reaction was continued for 10 min in the presence of inhibitors and stopped by adding 10 mL of 30% phosphoric acid. The stopped mixtures were spotted onto P81 paper and were washed with 10 mM Tris-HCl (pH 8.0) containing 0.1 M NaCl five times. The radioactivity of each spot was quantified with BAS imager. The inhibition studies of human EGFR tyrosine kinase activities were done using C-terminal human EGFR tyrosine kinase domain as described previously [26]. PYK2104 was synthesized as reported and used as a reference compound [26]. The concentration of inhibitor that gives 50% inhibition was designated as IC_50_ value.

#### Measurement of potential cytotoxicity

The cytotoxic activity was measured in vitro for the newly synthesized compounds using the SulfoRhodamine-B stain (SRB) assay using the method of Skehan [27]. Cells were plated in 96-multiwell microtiter plate (104 cells/well) for 24 h before treatment with the compound(s) to allow attachment of cell to the wall of the plate. Tested compounds were dissolved in DMSO and diluted with saline to the appropriate volume. Triplicate wells were prepared for each individual dose. Monolayer cells were incubated with the compound(s) for 48 h at 37 °C and in atmosphere of 5% CO_2_. After 48 h, cells were fixed, washed, and stained for 30 min with 0.4% (wt/vol) with SRB dissolved in 1% acetic acid. Unbound dye was removed by four washes with 1% acetic acid, and attached stain was recovered with Tris-EDTA buffer. Color intensity was measured in an ELISA reader. The relation between surviving fraction and drug concentration is plotted to get the survival curve for tumor cell line after the specified time.

## Figures and Tables

**Sch.1.**
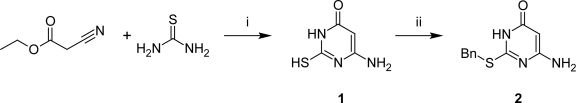
Reagents and conditions (i) NaOEt, reflux 3h; (ii) C_6_H_5_CH_2_Cl, DMSO/NaOH or DMF/K_2_CO_3_, r.t.

**Sch. 2.**
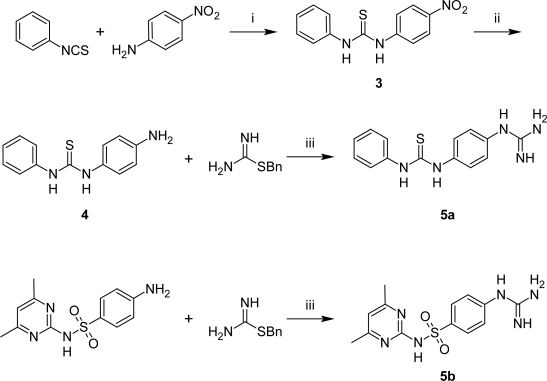
Reagents and conditions (i) KOH, EtOH, r.t.; (ii) MeOH, Zn, HCOONH_4_; (iii) EtOH, reflux 16h.

**Sch. 3.**
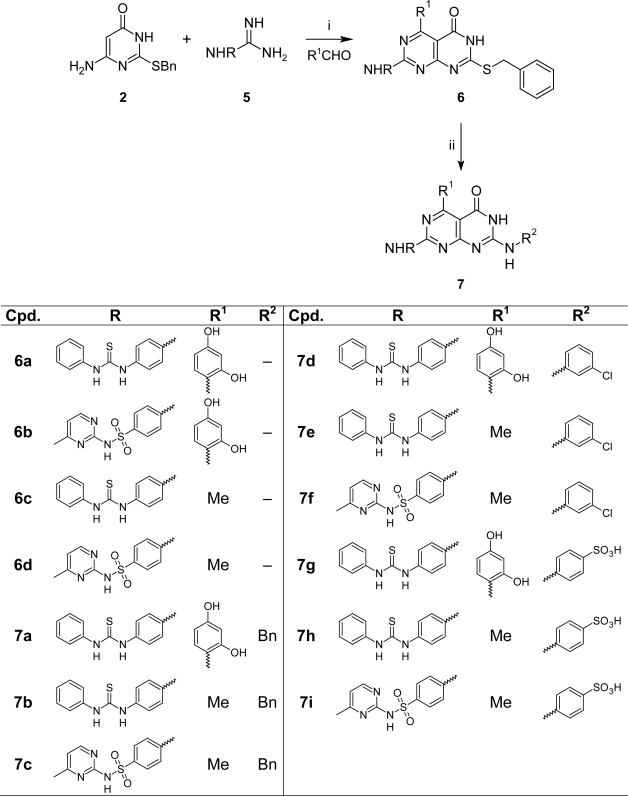
Reagents and conditions (i) ACOH, reflux 11h; (ii) different primary amines, DMF, reflux 8h.

**Tab. 1.** IC_50_ Values for the newly synthesized compounds as CDK inhibitors.

**Cpd.**	**IC_50_(μM)^a^**		
	**CDK2/CyclinA**	**CDK4/CyclinD**	**EGFR**
**6a**	5.0	>10	9.3
**6b**	2.80	2.14	5.5
**6c**	1.50	3.11	>10
**6d**	2.50	0.66	7.8
**7a**	0.31	3.33	>10
**7b**	0.86	4.85	>10
**7c**	2.60	>10	6.6
**7d**	>10	6.88	>10
**7e**	0. 25	2.55	4.9
**7f**	0.05	0.86	>10
**7g**	6.5	5.21	7.3
**7h**	6.0	>10	>10
**7i**	>10	>10	8.1
**Roscovitine**	0.5	–	–
**PYK2104**	–	–	0.0008

a At least two independent experiments were performed for each compound in order to determine IC_50_ in replicates and potency is expressed by the mean of IC_50_ values obtained by nonlinear regression analysis. Coefficient of variance /SD/ mean) ranges from 10 to 24%.

**Tab. 2.** Primary anti-cancer assay of the new compounds (Growth Inhibition %)

**Panel line**	**Cpd.^a^**	**6a**	**6b**	**6c**	**6d**	**7a**	**7b**	**7c**
	**Cells**							

**Non-Small Cell Lung Cancer**	A549/ATCC			42.13	21.19	58.41		64.44
	EKVX					36.79	28.5	
	HOP-92	102.6			26.97		20.79	

**Breast Cancer**	BT-549		29.05					
	HS 578T						33.65	
	MCF7						43.46	
	MDA-MB-231/ATCC			23.08			31.53	
	T-47D	26.93						23.41

**Ovarian Cancer**	IGROV1		30.74	34.33		29.74		61.71
	OVCAR-8		38.74					

**Leukemia**	HL-60(TB)			23.97			24.48	23.13
	K-562		20.12	27.89			53.68	32.76
	MOLT-4				43.38	36.7	32.94	22.58
	RPMI-8226			32.99		22.62	50.23	50.67

**Renal Cancer**	RXF 393	33.82		124.01				
	UO-31					21.35		39.48

**Melanoma**	UACC-257			25.23		60.26		71.36

**CNS Cancer**	SF-539			36.74			42.05	
	SNB-75							43.14

**Panel line**	**Cpd.^a^**	**7d**	**7e**	**7f**	**7g**	**7h**	**7i**	
	**Cells**							

**Non-Small Cell Lung Cancer**	A549/ATCC	44.71		51.98			39.12	
	EKVX							
	HOP-92		39.6	34.82	29.05	24.32		

**Breast Cancer**	BT-549					38.98		
	HS 578T		32.93				26.23	
	MCF7				33.32			
	MDA-MB-231/ATCC	21.44		30.36				
	T-47D						30.15	

**Ovarian Cancer**	IGROV1	39.7		54.43		28.01		
	OVCAR-8	26.8		21.76		50.24		

**Leukemia**	HL-60(TB)		42.9	21.6		28.97		
	K-562		28.19	26.48	20.11			
	MOLT-4		35.05			20.8		
	RPMI-8226	30.45	42.84	58.92		52.49		

**Renal Cancer**	RXF 393		112.9					
	UO-31	30.71		40.29		21.08	30.55	

**Melanoma**	UACC-257	79.49		57.86	32.66			

**CNS Cancer**	SF-539		23.47					
	SNB-75			28.53			23.21	

The newly synthesized compounds were evaluated in primary anti-cancer assay at 10^−5^M concentration against 60 human tumors cell line then the active compounds were tested at 5 differentconcentrations against the same 60 human tumor cell lines.

## References

[1] Sherr C (1996). Cancer cell cycles. Science.

[2] Sherr C (2000). The Pezcoller lecture: cancer cell cycles revisited. Cancer Res.

[3] Nigg EA (2001). Mitotic kinases as regulators of cell division and its checkpoints. Nat Rev Mol Cell Biol.

[4] Gould KL, Woodgett J (2000). Cyclin-dependent protein kinases. Front Mol Biol.

[5] Clark A (1996). Natural products as a resource for new drugs. Pharm Res.

[6] Melik-Ogandzhanyan RG, Khachatryan VE, Gapoyan AS (1985). Furo-, Thieno-, and Pyrrolo-[2,3-d]pyrimidines. Russ Chem Rev.

[7] Rewcastle GW, Bridge AJ, Fry DW, Rubin JR, Denny WA (1997). Tyrosine Kinase Inhibitors. 12. Synthesis and Structure−Activity Relationships for 6-Substituted 4-(Phenylamino)pyrimido[5,4-d]pyrimidines Designed as Inhibitors of the Epidermal Growth Factor Receptor. J Med Chem.

[8] Fry DW, Becker MA, Switzer RL (1995). Inhibition of human 5-phosphoribosyl-1-pyrophosphate synthetase by 4-amino-8-(beta-D-ribofuranosylamino)-pyrimido[5,4-d]pyrimidine-5′- monophosphate: evidence for interaction at the ADP allosteric site. Mol Pharm.

[9] Gready JE, McKinlay C, Gebauer MG (2003). Synthesis of quaternised 2-aminopyrimido[4,5-d]pyrimidin-4(3H)-ones and their biological activity with dihydrofolate reductase. Eur J Med Chem.

[10] Sanghvi YS, Larson SB, Matsumoto SS, Nord LD, Smee DF, Willis RC, Avery TH, Robins RK, Revankar GR (1989). Antitumor and antiviral activity of synthetic alpha- and beta-ribonucleosides of certain substituted pyrimido[5,4-d]pyrimidines: a new synthetic strategy for exocyclic aminonucleosides. J Med Chem.

[11] Tenser RB, Gaydos AK, Hay A (2001). Inhibition of Herpes Simplex Virus Reactivation by Dipyridamole. Antimicrob Agents Chemother.

[12] De la Cruz JP, Carrasco T, Ortega G, Sanchez De la Cuesta F (1992). Inhibition of ferrous-induced lipid peroxidation by pyrimido-pyrimidine derivatives in human liver membranes. Lipids.

[13] Sharma P, Rane N, Gurram VK (2004). Synthesis and QSAR studies of pyrimido[4,5-d]pyrimidine-2,5-dione derivatives as potential antimicrobial agents. Bioorg Med Chem Lett.

[14] Ram VJ, Goel A, Sarkhel S, Maulik PR (2002). A Convenient Synthesis and Hepatoprotective Activity of Imidazo[1,2-c]pyrimido[5,4-e]pyrimidine, Tetraazaacenaphthene and Tetraazaphenalene from Cyclic Ketene Aminals Through Tandem Addition-Cyclization Reactions. Bioorg Med Chem.

[15] Diaa AI, Amira ME, Elham EA (2009). Structure-based design of a new class of highly selective pyrazolo[3,4-d]pyrimidines based inhibitors of cyclin dependent kinases. ARKIVOC.

[16] Diaa AI (2009). Synthesis and biological evaluation of 3,6-disubstituted [1,2,4]triazolo[3,4-b][1,3,4]thiadiazole derivatives as a novel class of potential anti-tumor agents. Eur J Med Chem.

[17] http://www.rcsb.org/pdb/explore.do?structureId=1ke6

[18] (2003). Discovery Studio 20.

[19] Biginelli P (1893). [Aldureides of ethylic acetoacetate and ethylic oxalacetate]. Gazz Chim Ital.

[20] Roland KR (1956). Potential Purine Antagonists. I. Synthesis of Some 4,6-Substituted Pyrazolo [3,4-d] pyrimidines. J Am Chem Soc.

[21] Qin Y, Jian F, Jiang M, Yang X (2008). 1-(2-Nitrophenyl)-3-phenylthiourea. Acta Crystallogr Sect E Struct Rep Online.

[22] Meijer L, Borgne A, Mulner O, Chong JP, Blow JJ, Inagaki N, Inagaki M, Delcros JG, Moulinoux JP (1997). Biochemical and cellular effects of roscovitine, a potent and selective inhibitor of the cyclin-dependent kinases cdc2, cdk2 and cdk5. Eur J Biochem.

[23] Davies TG, Tunnah P, Meijer L, Marko D, Eisenbrand G, Endicott JA, Noble ME (2001). Inhibitor Binding to Active and Inactive CDK2: The Crystal Structure of CDK2-Cyclin A/Indirubin-5-Sulphonate. Structure.

[24] Cyril B, Philipp K (2006). Cdk2 and Cdk4 cooperatively control the expression of Cdc2. Cell Div.

[25] Oh CH, Kim HK, Lee SC, Oh C, Yang BS, Rhee HJ, Cho JH (2001). Synthesis and biological properties of C-2, C-8, N-9 substituted 6-(3-chloroanilino) purine derivatives as cyclin-dependent kinase inhibitors. Part II. Arch Pharm.

[26] Lee JY, Park YK, So I-S, Chung HK, Yang BS, Lee SJ, Park H, Lee YS (2001). 1,4-dioxane-fused 4-anilinoquinazoline as inhibitors of epidermal growth factor receptor kinase. Arch Pharm.

[27] Skehan P, Storeng R, Scudiero D, Monks A, McMahon J, Vistica D, Warren JT, Bokesch H, Kenney S, Boyd MR (1990). New Colorimetric Cytotoxicity Assay for Anticancer-Drug Screening. J Natl Cancer Inst.

